# Transfusion Practices in the Management of Sickle Cell Disease: A Survey of Florida Hematologists/Oncologists

**DOI:** 10.5402/2012/524513

**Published:** 2012-12-12

**Authors:** Levette N. Dunbar, LaRae Coleman Brown, Donna R. Rivera, Abraham G. Hartzema, Richard Lottenberg

**Affiliations:** ^1^Division of Hematology/Oncology, Department of Pediatrics, University of Florida, Box 100296, Gainesville, FL 32610, USA; ^2^Division of Hematology/Oncology, Department of Medicine, University of Florida, Box 100278, Gainesville, FL 32610, USA; ^3^Department of Pharmaceutical Outcomes and Policy, University of Florida, Box 100496, Gainesville, FL 32610, USA

## Abstract

The purpose of this study was to characterize transfusion practices in the management of sickle cell disease and to identify factors attributing to differences in prescribing practices among Florida hematologists/oncologists. A cross-sectional study was performed in 2005-2006 utilizing a mail survey. The survey instrument addressed practice characteristics, sickle cell patient populations, transfusion settings, indications and techniques, red blood cell phenotype specifications/modifications, use of practice guidelines, and educational resource utilization. One hundred fifty two physicians (75% adult-oriented, 25% pediatric) completed the survey. Non-academic practice settings (78 %) were the primary location. Pediatric practices had a larger percentage of patients with overt strokes, and receiving hydroxyurea therapy than adult-oriented practices. The majority of survey respondents did not request limited phenotypically matched red blood cells on a routine basis. The majority of pediatric practices (60%) had individually defined transfusion practice guidelines in contrast to 8% of adult-oriented practices. There were statistically significant differences for pediatric and adult-oriented practices in managing certain acute and chronic transfusion indications. Analysis of clinical vignette data revealed variation among hematologists/oncologists in the transfusion management of common clinical scenarios. The study underscores the need for the development and dissemination of comprehensive sickle cell transfusion guidelines and protocols.

## 1. Introduction

Red blood cell (RBC) transfusions are a mainstay of treatment in the management of patients with sickle cell disease (SCD). The majority of patients receive a transfusion during their lifetime. Indications for acute and chronic transfusion have expanded, resulting in increased numbers of patients receiving multiple transfusions [1]. Overall, there is a paucity of clinical trials directly addressing the use of red blood cell transfusions, and published prospective studies have focused primarily on patients with hemoglobin SS and not the other genotypes. All of the published randomized control trials (RCTs) have enrolled patients with hemoglobin SS. A RCT with a limited number of subjects demonstrated no benefit for prophylactic transfusion in pregnancy compared to transfusion at a target hemoglobin level in terms of maternal and perinatal complications [2]. Another RCT comparing conservative to aggressive transfusion regimens in elective surgery demonstrated equivalent efficacy [3]. The Stroke Prevention Trial in Sickle Cell Anemia (STOP) demonstrated efficacy of chronic transfusion compared to conventional therapy for the primary prevention of stroke in children at increased risk based on transcranial Doppler (TCD) velocity [4, 5]. Vichinsky et al. demonstrated a reduction in the rate of alloimmunization from 3% to 0.5% per unit and a reduction in hemolytic transfusion reactions by 90% with the use of limited phenotypic RBC matching in the STOP trial [6]. Recently, results from a RCT comparing preoperative transfusion use and no transfusion in patients with hemoglobin SS demonstrated increased complications for patients undergoing low- or moderate-risk procedures not receiving transfusions [7].

Guidelines based on review of literature, consensus or expert opinion pertaining to a broader range of clinical indications for transfusion have been published [8–12]. Uptake of recommendations from the published literature into clinical practice has not been examined.

In a survey of blood bank management of sickle cell patients at Comprehensive Sickle Cell Centers in the USA, a lack of consensus on transfusion practices for SCD was observed [13]. The study focused on transfusion service physicians and laboratory directors and did not address preferences of hematologists. The purpose of this study was to examine transfusion practices by hematologists/oncologists in the clinical management of patients with SCD.

## 2. Methods

A comprehensive literature search was performed to identify articles addressing transfusion management in SCD and to construct items, both demographic and clinical, for inclusion in the survey. A draft survey was generated with input from a health service researcher with expertise in physician surveys, a transfusion medicine specialist, and hematologists with expertise in SCD. The survey was refined with the input from a group of academic hematologists in other regions of the United States with particular interest in SCD. Face and content validity were evaluated by pilot testing the survey with current fellows and previous trainees of the University of Florida Hematology/Oncology fellowship program in practice locations outside of Florida. Feedback was obtained and revisions were implemented accordingly, with additional input from two other healthcare provider survey experts. The survey and an accompanying cover letter were submitted and approved by the Institutional Review Board.

The self-administered mail questionnaire used in this survey contained thirty-two (32) items. Items addressed include educational background, clinical practice setting, professional practice characteristics of physicians, their SCD patient populations, and transfusion practices (see survey, Appendix). We asked about the availability of information on transfusion recommendations for SCD patients, attendance at continuing education programs, and utilization of the National Institutes of Health (NIH) sickle cell disease management monograph available at the time of the survey. Selected items were multiple choice, while others were open ended or had an open-ended option attached. The survey included 4 clinical vignettes, where physicians were asked to choose their single most likely treatment recommendation. Questions regarding transfusion practices covered the use of phenotypically matched RBCs. RBC product modification choices were indicated as leukocyte reduced, irradiated, washed, sickle cell negative, and other products. Availability and frequency of automated exchange transfusion and transfusion recommendations for acute and chronic complications of SCD were also probed. There were 6 acute transfusion indications and 10 chronic transfusion indications, where practitioners were asked to rank responses using a Likert scale, using the following choices: always, sometimes, rarely, or never.

### 2.1. Study Design

The survey was mailed to hematologists/oncologists (H/Os) in the state of Florida from fall 2005 through spring 2006. A list of respondents was compiled from membership directories of the American Society of Hematology (ASH), the American Society of Clinical Oncology (ASCO), and the Florida Association of Pediatric Tumor Programs (FAPTP). Surveys were distributed to physicians in three sequential mailings, with telephone and email follow-up for nonresponders.

### 2.2. Survey Data Analysis

Data were input and organized for evaluation in Access 2007. SAS Version 9.1 (SAS Institute Inc., Cary, NC, USA) statistical software was used for all statistical analyses. Frequencies and percentages were calculated for categorical data, with means and standard deviations calculated for numerical data. Chi-square and Fisher's exact tests were used to test relationships between bivariate categorical data. Groups were compared using Mantel-Haenzel tests. *P* values < 0.05 were considered significant.

## 3. Results

One hundred fifty-two H/Os completed the survey out of 471 requested providers (32% response rate). The study's response rate may be deflated because not all practitioners contacted were involved in the treatment or transfusion of SCD patients in their practices. There was no ability to determine the reason for the nonresponse and whether this was related to area of practice or another factor. Data extraction from Medicaid claims revealed that only 264 practitioners in Florida provided RBC transfusions to SCD patients during the time period of the survey (Abraham Hartzema, unpublished data).

### 3.1. Survey Respondent and Clinical Practice Characteristics

The H/Os were asked to select pediatric or adult-oriented and academic or nonacademic as a practice setting. Eighty-two percent of the adult-oriented practice respondents were in a non-academic setting compared to 51% of the pediatric practice respondents. Ninety-three percent of the non-academic adult-oriented practitioners were greater than four years from the completion of their fellowship in comparison to the 86% of non-academic pediatric practitioners. Seventy-seven percent of the adult academic respondents and 69% of pediatric academic respondents were greater than four years postfellowship training. Thus, overall the majority of respondents completed fellowship training 4 or more years prior to the survey and were in a non-academic practice setting.

SCD practice pattern information is presented in Table 1 comparing pediatric and adult-oriented practitioners. Adult-oriented practice respondents reported having fewer SCD patients than pediatric H/Os. When comparing academic and non-academic practice respondents, 49% of the academic respondents had 51 or more SCD patients in comparison to only 17% of the non-academic practice respondents. The highest numbers of SCD patients with stroke were noted in the pediatric practices with 87% having six or more patients. Sixty-five percent of the adult practice respondents indicated no stroke patients. Fifty-four percent of the non-academic practice respondents had no stroke patients in comparison to 24% of the academic practice respondents. The majority of pediatric practice respondents (95%) had three or more patients receiving HU, with 40% having 16 or more patients receiving HU. In contrast, 70% of the adult practice respondents had two or fewer patients on HU, with 32% having no patients receiving HU. Sixty-two percent of academic practice respondents had three or more patients receiving HU, while 57% of the non-academic practice respondents had two or fewer patients receiving HU.

### 3.2. Transfusion Techniques

Most of the pediatric practice respondents (63%) routinely request limited or extended phenotype matching in comparison to only 30% of the adult practice respondents. Automated exchange transfusion was available on an emergent basis in the majority of pediatric (94%) and adult-oriented practices (70%).

**Figure 1:**
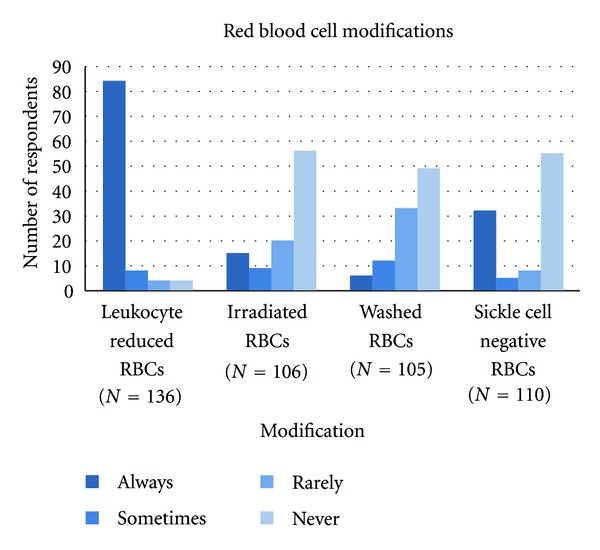
Red blood cell product requests excluding patients with bone marrow transplantation.

We asked respondents how often specific RBC products were requested for a sickle cell patient excluding the bone marrow transplantation setting. The results are summarized in Figure 1. Overall, respondents “always” requested leukocyte reduced products (84%). A minority of practitioners requested sickle negative RBCs all the time. Requests for irradiated products were limited as well. The majority never or only rarely requested washed blood products.

### 3.3. Transfusion Indications

#### 3.3.1. Acute Transfusion

**Figure 2:**
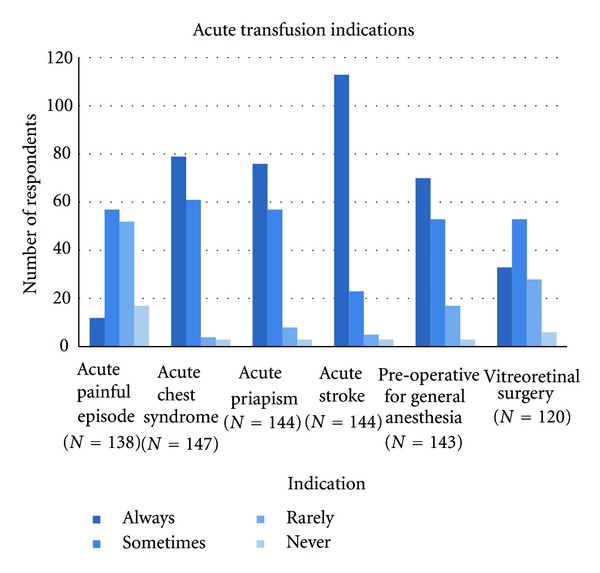
Acute transfusion indications for children and adults with Hb SS.

 We asked respondents what clinical indications they would consider for acute (episodic) transfusion in patients with SCD. The results are shown in Figure 2. Other acute indications were specified by respondents as write-in answers and included aplastic crisis, bone marrow transplant, severe symptomatic anemia, multiorgan system failure, hepatic sequestration, hospitalization for infection in a nonacute pain crisis, pregnancy with and without complications, nonhealing ulcers, and hypoxia with pneumonia. Overall 79% of the respondents thought that acute transfusion was “always” indicated for acute stroke. Fifty-four percent of respondents would “always” transfuse acutely for acute chest syndrome (ACS). Fifty-three percent would “always” transfuse for acute priapism. In addressing the pre-operative setting with general anesthesia, 49% would “always” and another 37% would “sometimes” transfuse red blood cells. Interestingly, 9% of respondents thought transfusion was “always” and 41% “sometimes” indicated for acute painful episodes. The majority of respondents (72%) indicated “always” or “sometimes” for vitreoretinal surgery.

Statistically significant differences among pediatric and adult-oriented practice respondents in reference to acute transfusion indications were identified. The following differences were observed for indications, where there is greater consensus in the literature [8–12, 14]. Pediatricians “rarely” considered acute painful episodes an indication for transfusion in comparison to adult practice respondents (78% versus 26%) (*P* < 0.0001). In contrast, 53% of adult practice respondents “sometimes” considered acute painful episode as an indication for transfusion compared to 3% of pediatric practice respondents (*P* < 0.0001). Pediatric practice respondents were more likely to report an acute stroke as an indication for acute transfusion with 95% reporting “always” in comparison to 73% of adult practice respondents (*P* < 0.005). General anesthesia was “always” an indication for transfusion in 73% of pediatric practices compared to 41% of adult practices (*P* = 0.003).

#### 3.3.2. Chronic Transfusion

**Figure 3:**
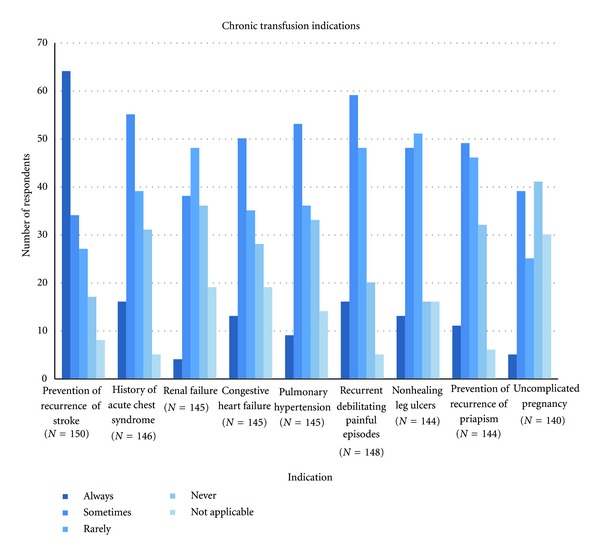
Chronic transfusion indications for children and adults with sickle cell disease.

 We also addressed clinical indications for chronic transfusion in patients with sickle cell disease. The results are shown in Figure 3. Other indications specified by respondents as written in answers included abnormal TCD, bone marrow failure, a fall in reticulocyte count, and viral infection.

We queried transfusion preferences for primary stroke prevention, but the survey question did not specifically address patients with hemoglobin SS or whether patients had a high risk TCD result. Therefore, it was not possible to discern the preferences of the respondents pertaining to primary stroke prevention, and thus the data are not shown. Prevention of stroke recurrence was “always” an indication for chronic transfusion in 95% of pediatric practice respondents, compared to only 25% of the adult practice respondents (*P* < 0.0001). A history of ACS was “always” an indication for chronic transfusion in 11% of all respondents. A history of ACS was “sometimes” an indication for chronic transfusion in 68% of pediatric and 28% of adult practice respondents (*P* < 0.0001). The occurrence of recurrent debilitating painful episodes was “always” indicated by a minority of respondents. Recurrent debilitating pain was “sometimes” an indication for chronic transfusion for 60% of pediatric and 33% of adult-oriented practice respondents (*P* = 0.005) There was a spectrum of responses pertaining to transfusion for uncomplicated pregnancy with 56% of respondents selecting “never” or “rarely.” Although the majority (71%) of the pediatric respondents selected “N/A,” there was no statistically significant difference between pediatric and adult practice respondents. There was no statistically significant difference between pediatric and adult practice respondents for congestive heart failure, pulmonary hypertension, nonhealing ulcers, or recurrent priapism, where the majority indicated sometimes or rarely.

#### 3.3.3. Clinical Vignettes

**Figure 4:**
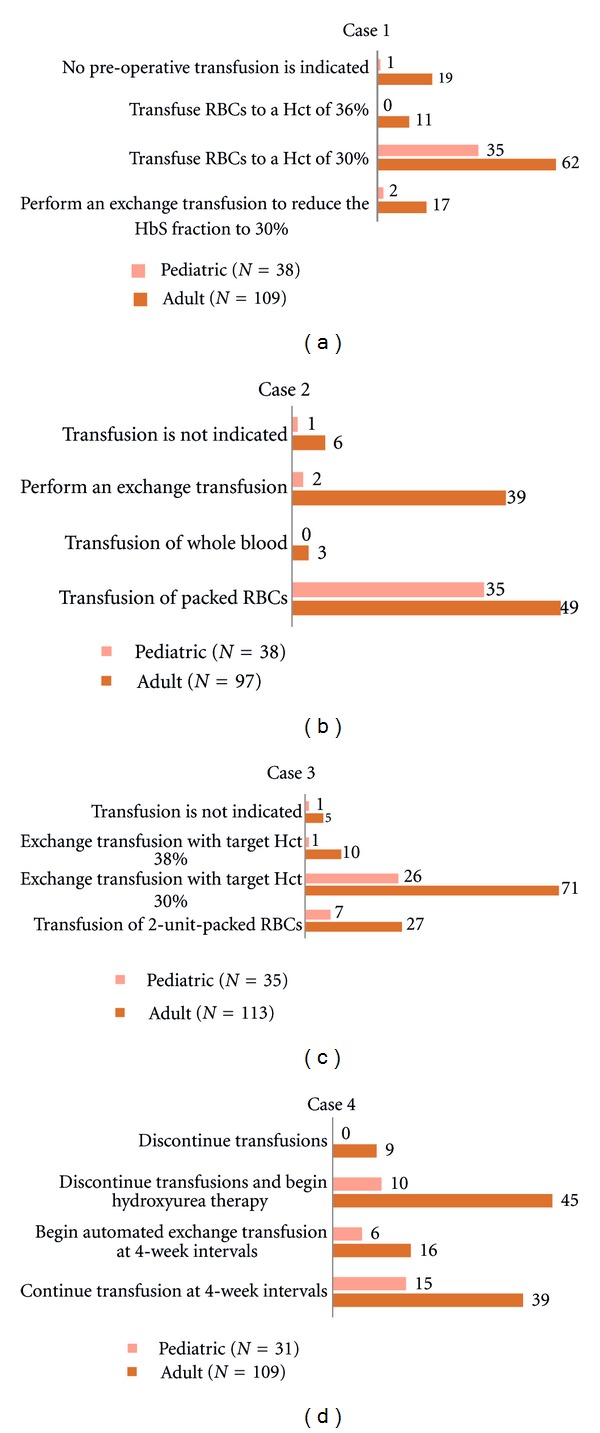
Transfusion practices assessed by responses to clinical vignettes (Cases 1–4).

The clinical vignettes were based on commonly encountered scenarios involving the transfusion management of SCD patients (see Table 2). The vignettes revealed statistically significant differences between pediatric and adult choices (Figure 4). In case 1, the vast majority of pediatric respondents indicated simple transfusion to a hematocrit of 30% for an adolescent undergoing cholecystectomy. Although representing a minority of the adult-oriented respondents, there were responses opting for no transfusion (17%) or an exchange transfusion (16%), respectively. A marginal number of adult practice respondents (10%) indicated the less desirable option of a higher target hematocrit (Hct) level, whereas none of the pediatric respondents opted for this choice [15]. In case 2, regarding splenic sequestration, the majority of pediatric respondents selected simple transfusion, which is appropriate given the low hemoglobin level; however, 40% of the adult-oriented practice respondents indicated exchange transfusion. The majority of respondents indicated utilizing exchange transfusion with an appropriate target Hct level for Case 3 with progressive ACS [14, 15].

 Case 4 addressed duration of chronic transfusions in a patient with stroke occurring in childhood who is now an adult. Continuing simple transfusions was the mainstay consideration by both pediatric and adult respondents at 48% and 36%, respectively. Exchange transfusion was indicated by a minority of respondents. While both groups also gave consideration to the option of discontinuing transfusion, all pediatric practice respondents selecting discontinuation of transfusions would prescribe HU therapy (32% of responses) for this young adult, whereas simple discontinuation of transfusions alone was selected by 8% of adult respondents.

#### 3.3.4. Guidelines and Educational Resources

There was a statistically significant difference between adult and pediatric practice respondents with respect to the use of transfusion guidelines and the use of the NIH Management of SCD monograph [11]. When asked if their practice used specific transfusion guidelines, 60% of pediatric practice respondents answered “yes” compared to only 8% of adult-oriented practice respondents (*P* < 0.0001). Fifty-five percent of pediatric practice respondents reported use of the NIH monograph compared to 26% of adult-oriented practice respondents (*P* = 0.0012).

 Physicians were asked if information was readily available on transfusion management of SCD patients. Eighty-two percent of pediatric-oriented and 63% of the adult-oriented practice respondents indicated availability. Interestingly through a closer examination, academic adult and pediatric practice respondents both had 88% availability, whereas their non-academic counterparts accounted for the observed differences. Seventy-one percent of the pediatric and nineteen percent of the adult-oriented practice respondents had attended at least one conference or presentation on the management of SCD (not specifying transfusion as a topic) in the two-year period of time prior to the survey; there was also a significant difference comparing academic to non-academic practices (61 versus 24%), respectively, (*P* < 0.0001). Importantly, in response to an open-ended question about materials that would be helpful, 35% of physicians who requested materials wanted specific transfusion guidelines for the management of SCD.

## 4. Discussion

Florida has one of the largest SCD populations in the United States [16]. The findings of the survey provide insights into transfusion practices across a spectrum of clinical practice settings. The results of the survey indicate variation in the transfusion management of SCD among Florida H/Os. Although the non-academic and adult-oriented practices are largest in number, the academic and pediatric practices have the most SCD patients and more readily use resources available for SCD management. However, comparison of consensus-based recommendations and transfusion practices of respondents revealed variability between recommended and typical practice patterns. There are factors at the patient, provider, and health care delivery system levels which contribute to medical decisions concerning transfusion not captured by this type of survey.

Phenotype matching and blood product selection are important aspects of transfusion management of patients with SCD. In a survey of 1182 North American laboratories, the majority did not determine the red cell antigen phenotype of nonalloimmunized SCD patients beyond ABO and D [17]. In the assessment of blood banks associated with institutions previously designated as Comprehensive Sickle Cell Centers, more than 90 percent of institutions provided C, E, and Kell routinely [13]. In this study, the majority of respondents did not routinely request phenotypically matched RBCs for SCD patients until the patient demonstrated a new antibody. Of note, pediatric practice respondents did request up-front matching more frequently than the adult-oriented practices. The majority of respondents used leukocyte reduced products, consistent with current recommendations [6, 11]. There was variability in the request of sickle negative products, which was consistent with previous survey results of the blood bank practices [17]. However, a survey of blood bank centers associated with previously designated Comprehensive Sickle Cell Centers revealed 66% of respondents always provided sickle cell trait-negative RBCs [13]. The indications for washed and irradiated RBC product are limited to specific situations such as previous anaphylactic transfusion reactions or bone marrow transplant recipients, respectively. The survey question was specifically indicated to address choices in an SCD patient who had not had a bone marrow transplant. Nevertheless, 15% of physicians responded they “always” request irradiated blood and 6% responded that they “always” request washed blood. This response rate is in contrast to the survey of blood banks, where irradiated and washed RBCs were rarely or never indicated by the majority of institutions [13]. The discrepancies found in this portion of the survey highlight specific provider issues, which can be addressed in providing appropriate blood products for SCD patients.

Our survey addressed clinical scenarios, where routine transfusion would not be indicated. A small RCT previously demonstrated no benefit of prophylactic transfusion in pregnant patients with hemoglobin SS in terms of maternal and perinatal complications [2]. In concordance with these findings, the majority of survey respondents did not routinely transfuse for uncomplicated pregnancy. Published recommendations do not identify acute painful episodes without complications as an indication for transfusion [10, 12, 14]. In our survey, 9% of the sample indicated this was “always” an indication, whereas the combination of rarely and never constituted 50% of the responses. There is a clear opportunity to provide practitioners with information on when blood transfusions would not be an indicated intervention, as acute painful episodes are a common complication of SCD.

Cholecystectomy is the most commonly performed surgery in patients with SCD [18]. In the clinical vignette case 1, the majority of adult and pediatric respondents indicated conservative transfusion management of laparoscopic cholecystectomy with an appropriate end point hematocrit level [14, 15]. Variance in the responses for adult-oriented practitioners compared to the pediatric practice respondents was similarly reflected in the more generic preoperative clinical scenario queried in the survey section on acute transfusion indications. A contemporary survey of North American members of the Society for Pediatric Anesthesia using clinical vignettes showed preference for preoperative transfusion for patients with increasing sickle-cell-related severity and invasiveness of procedures, but there was a considerable variability of responses [19]. The results of a recent RCT showing the benefit of preoperative transfusion compared to no transfusion in patients undergoing cholecystectomy and other moderate risk procedures was not available at the time of the current or previously mentioned survey [7].

The majority (95%) of respondents selected to use transfusion “always” or “sometimes” for ACS which is consistent with the findings of physicians participating in the National Acute Chest Syndrome Study [20]. There are limited data addressing transfusion use in vitreoretinal surgery and most of the reports are dated [21]. It is not surprising that responses for this clinical indication were varied. In summary, for most of the acute indications, no clinical trial data are available to provide strong recommendations.

RCT data support chronic transfusions for the primary prevention of stroke in high risk children [4, 5]. Unfortunately, our survey question did not explicitly indicate a prerequisite for high risk TCD results, and we were unable to infer how the sample frame would have handled this indication. Observational data support indefinite transfusion for secondary stroke prevention in children [22]. Ninety-five percent of pediatric practice respondents selected “always”, in comparison to 25% of adult-oriented practice respondents. Multiple factors may account for this difference and may include familiarity with the apparent risk for event recurrence beyond childhood years and the lack of availability for an oral iron chelator at the time of the survey [21]. Furthermore, chronic transfusion practices in the adult SCD population may pose logistical difficulties for the physician and the patient.

Patients with higher baseline hemoglobin/hematocrit levels pose challenges when transfusions are indicated to reduce the fraction of Hb S containing cells. Limited data are available to draw upon in addressing the transfusion of patients with Hb SC or HbS/beta+ thalassemia. In the clinical vignette case 3, for a patient with Hb SC presenting with ACS, the majority of respondents selected exchange transfusion. Also in the survey, the majority of respondents indicated having emergent availability of apheresis to perform a red cell exchange. Although no clinical trial has examined simple techniques compared to exchange techniques in ACS, there is literature supporting effectiveness of red cell exchange [20, 23, and 24]. A published guideline for treatment of ACS using a clinical severity score has incorporated both techniques [23]. In this particular case, the high hematocrit level would preclude using simple transfusion, selected by approximately 20% of the respondents.

 The last clinical vignette addressed a typical scenario pertaining to patients aging out of pediatric practices. Interestingly, there were just as many physicians that opted to “discontinue transfusions and begin HU” as there were to “continue transfusions”. At the time of the survey, use of HU for this indication was derived from limited observational data published on pediatric patients with previous strokes [24]. The Stroke With Transfusion Changing to Hydroxyurea (SWiTCH) trial was designed to address this issue in children; at the time of the survey, there was no prospective clinical study data to inform decision making [25].

There are limitations to this physician survey. As a result of nonresponse bias, the completed responses may not be a true representation of a larger sample. Although we attempted to specifically contact only H/Os prescribing transfusions to SCD patients, our sample size of 474 represents all physicians listed in the directories and not actually the number of H/Os managing SCD patients with transfusions. Generalization of the results is also limited by the fact that only one state was sampled and practice can vary by state and country based on perceived standard of care and available resources [26, 27]. This survey provided a cross-sectional evaluation of SCD transfusion practices during 2005-2006. The majority of results likely remain relevant to current practices. However, at the time of the survey, deferasirox was just undergoing Food and Drug Administration approval; therefore, deferoxamine was the only readily available iron chelating agent in the USA Followup surveys will be informative and necessary pertaining to chronic transfusion with the availability of an effective oral iron chelator. Our study indicates variability in the incorporation of literature-based approaches when comparing pediatric and adult-oriented practices. In addition, routine use of local transfusion guidelines and availability of exchange transfusion on an emergent basis were more widespread for pediatric providers. Interestingly, we also found more widespread use of HU therapy amongst pediatric providers.

Many points of reference for SCD treatment are considered controversial with the lack of extensive high-grade evidence and therefore differences in actual practice should be anticipated. Availability of comprehensive recommendations on the transfusion management of SCD in a user-friendly format would provide an opportunity to eliminate potentially unnecessary transfusions, enhance the proportion of patients who benefit from chronic transfusion, and reduce transfusion complications by making the most effective clinical decisions. The National Heart, Lung, and Blood Institute (NHLBI) is currently in the process of producing clinical guidelines for patients with sickle cell disease using rigorous methodology including systematic review of the literature and grading of evidence and recommendations [28]. There will be an opportunity to reexamine H/O practices in Florida after those guidelines are available. There remains a clear need for prospective clinical trials addressing blood transfusion in patients with SCD.

## Figures and Tables

**Table 1:** Sickle cell practice patterns among Florida hematologists/oncologists.

Number of SCD patients	Practice type	Yes number of respondents	Response rate	*P* value*
NONE	Adult	22	19.3%	0.0012
	Pediatric	0	0.00%	
1–10	Adult	72	63.2%	<0.0001
	Pediatric	1	2.6%	
11–50	Adult	18	15.8%	0.1544
	Pediatric	3	7.9%	
51 or more	Adult	2	1.8%	<0.0001
	Pediatric	34	89.5%	

Overall *P* value for group analysis (NONE, 1–10, 11–50, 51, or more) <0.0001.

**Table 2:** Clinical vignettes.

Case 1: A 16-year-old boy with sickle cell anemia (Hb SS) is scheduled for elective laparoscopic cholecystectomy. The baseline labs reveal that Hct is 22% and Hb is 7.2.	

Case 2: A 4-year-old girl with known sickle cell anemia (Hb SS) presents to the Emergency Department with a 12-hour history of abdominal pain, nausea, vomiting, and lethargy. Physical examination reveals an easily palpable and tender spleen. The CBC shows WBC 29,000/*μ*L with 80% neutrophils and 12% bands, and Hct 12%, and platelets 88,000/*μ*L. The physician in charge requests assistance in transfusion recommendations.	

Case 3: A 28-year-old woman with Hb SC disease has acute chest syndrome with progressive hypoxemia, despite oxygen supplementation. Review of the CBC reveals WBC 22,000/*μ*L, Hct 28%, and platelets 530,000/*μ*L.	

Case 4: A 21-year-old male with sickle cell anemia (Hb SS) would like to enter your practice. The patient has been undergoing transfusions of 2 units packed RBCs, every 4 weeks since a stroke at age 12 with a goal to maintain his Hb S level at ~50%. He has been on deferoxamine therapy over the past 7 years.	

## Appendix

Please circle the *best* answer, unless otherwise indicated.

### A. Background and Professional Practice

(A-1) Which of the following best describes the primary *location* of your current practice?
(1) Academic Medical Center
(2) Private Practice
(3) Other:_____________________
(A-2) Which of the following programs best describes your fellowship *training* experience?
(1) Oncology
(2) Hematology
(3) Hem/Onc
(4) Other:__________________________
(A-3) How many years has it been since you completed fellowship training?
(1) Less than 1
(2) 1–3
(3) 4–7
(4) 8–12
(5) 13 or more
(A-4) Which best describes your current *practice* situation?
(1) Adult hematology
(2) Adult oncology
(3) Adult hem/onc
(4) Pediatric hem/onc
(5) Adult & Peds hem/onc
(6) Other: _______________

### B. Sickle Cell Patient Population

(B-1) How many SCD patients do you currently have in your practice?
(1) NONE
(2) 1–5
(3) 6–10
(4) 11–15
(5) 16–20
(6) 21–50
(7) 51 or more
(B-2) What is the average number of SCD patients you see in a month?
(1) NONE
(2) 1-2
(3) 3–5
(4) 6–10
(5) 11–15 
(6) 16–20 
(7) 20 or more
(B-3) What is the *age range* (in years) of your SCD patients? (*Circle ALL that apply*)
(1) 16 or younger 
(2) 17–21
(3) 22–30
(4) 31 or older
(B-4) Of the sickle cell patients in your practice how many have experienced an overt *stroke*?
(1) NONE
(2) 1–5
(3) 6–10
(4) 11–15
(5) 6–20
(6) 21–50 
(7) 51 or more
(B-5) How many SCD patients do you have receiving *hydroxyurea*?
(1) NONE
(2) 1-2
(3) 3–5
(4) 6–10
(5) 11–15
(6) 16 or more

### C. Transfusion Practice

(C-1) Which best describes the *setting* used for the *nonemergent* transfusion of SCD patients? (*Circle ALL that apply*)

- Hospital as an inpatient
- Day hospital (including 23 hour observation)
- Hospital Oncology Infusion Center
- Office Oncology Infusion Center
- Other:______________________________
- Does your practice site have defined criteria (e.g., guidelines) for the transfusion management of SCD patients?
- (1) YES
- (2) NO

(C-3) Does your practice *routinely* request phenotypically matched RBC's for sickle cell patients?

1. Yes, Extended/Complete Matching
2. Yes, Limited Matching (e.g., antigens C, E, Kell)
3. No, not until the patient makes an antibody
4. Please indicate how often each of the following RBC products is requested for a sickle cell patient who has *not* had a bone marrow transplant.
  Blood Product	Always	Sometimes	Rarely	Never
  (a.) Nonleukocyte Reduced RBCs	3	2	1	0
  (b.) Leukocyte Reduced RBCs	3	2	1	0
  (c.) Irradiated RBCs	3	2	1	0
  (d.) Washed RBCs	3	2	1	0
  (e.) Sickle Cell negative RBCs	3	2	1	0
  (f.) Other Products (please specify):	3	2	1	0
5. Is Automated Exchange Transfusion (Apheresis) available *on an emergent basis* to your patients?

1. YES
2. NO
3. For Hb SS patients, when do you consider the following as an indication for *episodic (acute) transfusion*?
  	Always	Sometimes	Rarely	Never
  (a.) Acute Painful Episode	3	2	1	0
  (b.) Acute Chest Syndrome	3	2	1	0
  (c.) Acute Priapism	3	2	1	0
  (d.) Acute Stroke	3	2	1	0
  (e.) Pre-operative for General Anesthesia	3	2	1	0
  (f.) Vitreoretinal Surgery	3	2	1	0
  (g.) Other Indications (please specify):	3	2	1	0
4. For SCD patients, when do you consider the following as an indication for *chronic transfusion*?
  	Always	Sometimes	Rarely	Never
  (a.) Primary Prevention of Stroke	3	2	1	0
  (b.) Prevention of Recurrence of Stroke	3	2	1	0
  (c.) History of Acute Chest Syndrome	3	2	1	0
  (d.) Renal Failure	3	2	1	0
  (e.) Congestive Heart Failure	3	2	1	0
  (f.) Pulmonary Hypertension	3	2	1	0
  (g.) Recurrent Debilitating Painful Episodes	3	2	1	0
  (h.) Nonhealing Leg Ulcers	3	2	1	0
  (i.) Prevention of Recurrence of Priapism	3	2	1	0
  (j.) Uncomplicated Pregnancy	3	2	1	0
  (k.) Other indications (please specify):	3	2	1	0
5. Please indicate the number of SCD patients in your practice currently on a Chronic Transfusion Program, that undergo the therapies described below.
  Therapy	Number of Patients
  (a.) Simple Transfusion	
  (b.) Partial Manual Exchange Transfusion	
  (c.) Automated Exchange Transfusion	

### D. Iron Chelation: Deferoxamine (Desferal) Therapy

(D-1) What measures do you use to assess iron levels when *starting* deferoxamine therapy? (*Circle ALL that apply*)
(1) Steady State Serum Ferritin Levels
(2) Liver Biopsy
(3) Radiologic Imaging (MRI, CT)
(4) Number of RBC Units Transfused
(D-2) If you use *Serum Ferritin Levels* as an indication to begin deferoxamine therapy, what is your trigger value?

1. 1000 ng/mL
2. 1500 ng/mL
3. 2000 ng/mL
4. 2001 ng/mL or more
5. If you use the *Number of RBC Units transfused* as an indication to begin deferoxamine therapy, what is your trigger number?
6. (1) 0–19
7. (2) 20–29
8. (3) 30–39
9. (4) 40–49
10. (5) 50–59
11. (6) 60 or more
12. How many SCD patients in your practice have *indications* for deferoxamine?

1. NONE
2. 1-2
3. 3–5
4. 6–10
5. 11–15
6. 16–20
7. 21 or more
8. How many SCD patients in your practice are *prescribed* deferoxamine?
9. NONE
10. 1-2
11. 3–5
12. 6–10
13. 11–15
14. 16–20
15. 21 or more
16. Despite having an indication for deferoxamine, many patients are not receiving it. In your opinion, evaluate the importance of each of the following reasons for *NOT* using deferoxamine when clinically indicated?
  Reasons for Not Using Deferoxamine	Very Important	Somewhat Important	Not Important
  (a.) Patient declines	3	2	1
  (b.) Cost	3	2	1
  (c.) Lack of patient compliance to physician recommendations	3	2	1
  (d.) poor understanding by patient/family	3	2	1
  (e.) Lack of healthcare providers to supervise therapy	3	2	1
  (f.) Patient's fear of side effects	3	2	1
17. For SCD patients in your practice receiving deferoxamine, which of the following are routes of administration? (*Circle ALL that apply*)

1. Continuous subcutaneous infusion
2. Subcutaneous injection
3. Intravenously

### E. Transfusion Practices: Clinical Vignettes

Please circle the *SINGLE* most likely treatment you would recommend, based on your experience with SCD patients.
(E-1) A 16 y.o. boy with sickle cell anemia (Hb SS) is scheduled for elective laparoscopic cholecystectomy. The baseline CBC reveals Hct 22% and Hb 7.2.
*You recommend the following…*
(1) Perform an exchange transfusion to reduce the Hb S fraction to 30%
(2) Transfuse RBCs to a Hct of 30%
(3) Transfuse RBCs to a Hct of 36%
(4) No pre-operative transfusion is indicated
(E-2) A 4 y.o. girl with known sickle cell anemia (Hb SS) presents to the Emergency Department with a 12-hour history of abdominal pain, nausea, vomiting, and lethargy. Physical examination reveals an easily palpable and tender spleen. The CBC shows WBC 29,000/*μ*L with 80% neutrophils and 12% bands, Hct 12%, platelets 88,000/*μ*L. The physician in charge requests assistance in transfusion recommendations.
*You recommend the following…*
(1) Transfusion of packed RBCs
(2) Transfusion of whole blood
(3) Perform an exchange transfusion
(4) Transfusion is not indicated
(E-3) A 28 y.o. woman with Hb SC disease has acute chest syndrome with progressive hypoxemia, despite oxygen supplementation. Review of the CBC reveals WBC 22,000/*μ*L, Hct 28%, platelets 530,000/*μ*L.
*You recommend the following…*
(1) Transfusion of 2 units packed RBCs
(2) Exchange transfusion with target Hct 30%
(3) Exchange transfusion with target Hct 38%
(4) Transfusion is not indicated
(E-4) A 21 y.o. male with sickle cell anemia (Hb SS) would like to enter your practice. The patient has been undergoing transfusions of 2 units packed RBCs, every 4 weeks since a stroke at age 12 with a goal to maintain his Hb S level at ~50%. He has been on deferoxamine therapy over the past 7 years.
*What are your recommendations in regard to blood transfusions?*
(1) Continue transfusion at 4-week intervals
(2) Begin automated exchange transfusion at 4-week intervals
(3) Discontinue transfusions and begin hydroxyurea therapy
(4) Discontinue transfusions

### F. Educational Resources

(F-1) Information on transfusion of sickle cell patients is readily available to me.
(1) YES
(2) NO
(F-2) In the past two years, I have attended at least one conference or presentation on the management of sickle cell disease.
(1) YES
(2) NO
(F-3) I currently use The Management of Sickle Cell Disease monograph published by the National Institutes of Health (4th edition 2002), http://www.nhlbi.nih.gov/health/prof/blood/sickle/sc_mngt.pdf.
(1) YES
(2) NO
(F-4) What type of materials would be helpful in managing patients with sickle cell disease?
… … … …
